# Use of Standardized, Quantitative Digital Photography in a Multicenter Web-based Study

**Published:** 2009-01-12

**Authors:** Joseph A. Molnar, Wesley K. Lew, Derek A. Rapp, E. Stanley Gordon, Denise Voignier, Scott Rushing, William Willner

**Affiliations:** ^a^ Departments of Plastic and Reconstructive Surgery, Wake Forest University School of Medicine, Winston-Salem, NC; ^b^ Department of General Surgery, USC University Hospital, Los Angeles, CA; ^c^ Department of General Surgery, Beth Israel Deaconess Medical Center, Boston, MA; ^d^ Public Health Sciences, Wake Forest University School of Medicine, Winston-Salem, NC; ^e^ Medical Photography, Wake Forest University School of Medicine, Winston-Salem, NC

## Abstract

**Objective:** We developed a Web-based, blinded, prospective, randomized, multicenter trial, using standardized digital photography to clinically evaluate hand burn depth and accurately determine wound area with digital planimetry. **Methods:** Photos in each center were taken with identical digital cameras with standardized settings on a custom backdrop developed at Wake Forest University containing a gray, white, black, and centimeter scale. The images were downloaded, transferred via the Web, and stored on servers at the principal investigator's home institution. Color adjustments to each photo were made using Adobe Photoshop 6.0 (Adobe, San Jose, Calif). In an initial pilot study, model hands marked with circles of known areas were used to determine the accuracy of the planimetry technique. Two-dimensional digital planimetry using SigmaScan Pro 5.0 (SPSS Science, Chicago, Ill) was used to calculate wound area from the digital images. **Results:** Digital photography is a simple and cost-effective method for quantifying wound size when used in conjunction with digital planimetry (SigmaScan) and photo enhancement (Adobe Photoshop) programs. The accuracy of the SigmaScan program in calculating predetermined areas was within 4.7% (95% CI, 3.4%–5.9%). Dorsal hand burns of the initial 20 patients in a national study involving several centers were evaluated with this technique. Images obtained by individuals denying experience in photography proved reliable and useful for clinical evaluation and quantification of wound area. **Conclusion:** Standardized digital photography may be used quantitatively in a Web-based, multicenter trial of burn care. This technique could be modified for other medical studies with visual endpoints.

Modern technology, specifically the Internet and digital imaging, has made it possible to efficiently communicate clinical and research information between institutions. One of the most prominent uses has been in radiology. Digitization and Web-based diagnostic imaging systems have improved image capturing, data storage, and diagnostic yield, as well as providing significant cost savings.^1^–^4^ Goodwin et al^5^ described a “virtual craniofacial team” that utilized digital cameras and the internet. Vascular surgeons and ophthalmologists have also used telemedicine for classification and treatment of lower extremity wounds and diabetic retinopathy, respectively.^5^–^7^ While these techniques have been used successfully for qualitative evaluation of images, these techniques do not provide a standardized technique that may be successfully used for quantitative evaluation.

To properly evaluate a new treatment of acute hand burn injury, a prospective, randomized, controlled, blinded, multicenter trial was designed. One of the key endpoints of this study was to determine the amount of burn wound progression, requiring accurate evaluation of the size of the burn. As the study was designed to be Web based for rapid data evaluation by the principal investigator, it was deemed most efficient to collect the photos digitally. Prior investigators verified the reliability of digital imaging for the diagnosis of hand burns.^8^,^9^ As no method was found in the literature, a technique was developed to provide reproducible high-quality images suitable for quantitative evaluation, even when collected by inexperienced photographers. This technique uses commercially available software that can reliably determine surface area of wounds with digital planimetry and demonstrated a time and cost savings when compared with 35-mm film techniques.

This article discusses in detail the validation of the technique of digital photography only for the purpose of reproducibility for use in future studies. The results of the clinical study have been previously presented.^1^

## MATERIALS AND METHODS

**Figure 1:**
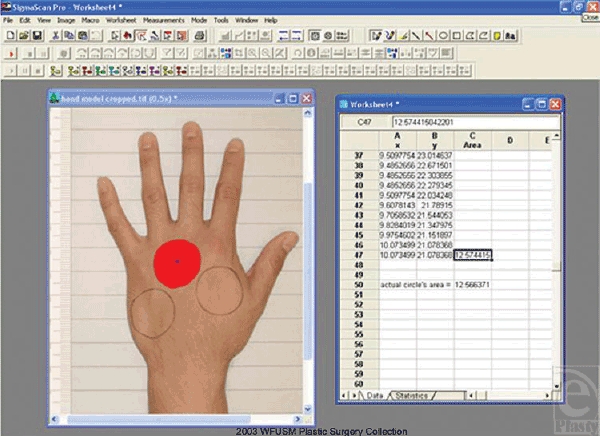
To determine the accuracy of the SigmaScan planimetry, wound models with known areas were created with circles of diameter 4 cm.

The techniques outlined were designed for a clinical study to evaluate a new technique of acute burn care.^1^ Since visual observation remains the “gold standard” to determine the size of the wounds, it was necessary to develop a quantitative and standardized technique of digital photography that would allow objective evaluation by digital planimetry. Before applying this technique clinically, it was necessary to validate the technique in the laboratory with a simulated wound model. Once this validation was completed, we could apply the technique in a prospective multicenter trial. To determine the accuracy of the planimetry technique, standard wound models of known size were created on a volunteer hand. The dorsa of 3 separate hands were marked with 3 circular templates of a predetermined area of 12.566 cm^2^ each circle. Care was taken to ensure that the final diameter on the hand was 4 cm. A circle shape was used as this is potentially one of the most difficult shapes to outline accurately with the planimetry techniques used. Images were obtained using the same method and custom backdrop described below. The model hand was repositioned and images were captured 4 times for 3 separate hands, for a total of 12 images. The digital planimetry program, SigmaScan Pro 5.0 (SPSS Science, Chicago, Ill), was then used to calculate the areas marked from each image (Fig 1). The sum of the 3 circular areas were calculated and then compared with the actual predetermined areas of 37.698 cm^2^ (=3 × 12.566 cm^2^). The data were analyzed using a *t* test.

Seven centers participated in the study. In the clinical portion of the study, images were photographed at each center with a Nikon Coolpix 995 digital camera (Nikon, Melville, NY) with standard settings for flash, white balance, and zoom as instructed on our Web site (www.burnvac.org). High-resolution photos were captured (9 MB), downloaded onto a PC in TIFF format, and sent through the Internet to a secure central server at the principal investigator's institution for analysis and storage. Patient confidentiality was maintained by appropriate firewalls, encoding the data, and using a code for each patient that was in no way related to identifying information such as patient hospital number.

**Figure 2:**
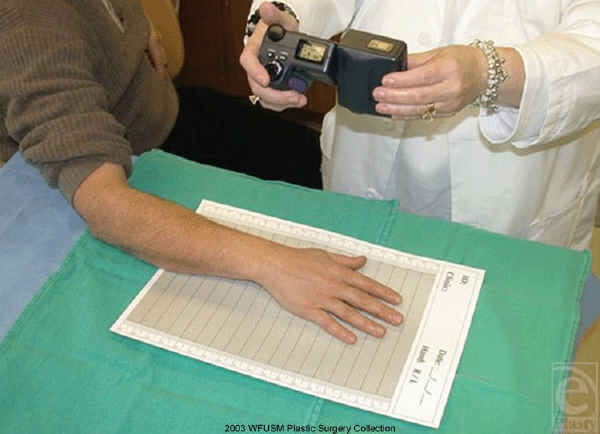
For validation of the technique, images were captured with the standardized background and digital camera in the manner to be used in the clinical study.

The dorsum of the hand was photographed over a reference backdrop developed by the Department of Plastic and Reconstructive Surgery in association with Medical Photography at Wake Forest University School of Medicine. The backdrop allows internal standardization for each photograph in both dimension and color. It is made of flexible posterboard, with gray, white, and black reference scales and marked with horizontal gradations 2 cm apart (Fig 2). The backdrops may be gas sterilized for use in the operating room or with acute open wounds.

**Figure 3:**
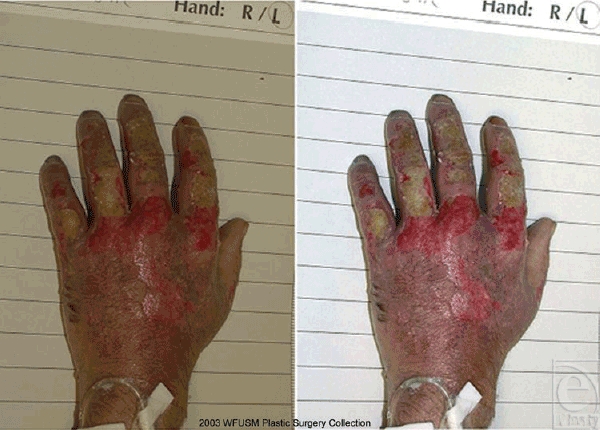
Digital image quality of a clinical image from the prospective multicenter trial before (left figure) and after (right figure) adjustment with Adobe Photoshop.

Digital images were evaluated on a 21-in Trinitron monitor (Sony Corporation, New York), calibrated with OptiCAL 3.5 (Pantone, Lawrenceville, NJ). Adobe Photoshop 6.0 (Adobe, San Jose, Calif) was used to compensate for any exposure or color differences found in the original digital files (Fig 3). Exposure and color corrections were made by setting a white point from a white area on the reference backdrop (image/adjust/levels/white eyedropper). Color was further refined by using the gray eyedropper to color neutralize the 18% gray portion of the reference backdrop (image/adjust/levels/gray eyedropper). In both cases, the eyedropper tool was set to sample a 5 × 5-pixel area.

**Figure 4:**
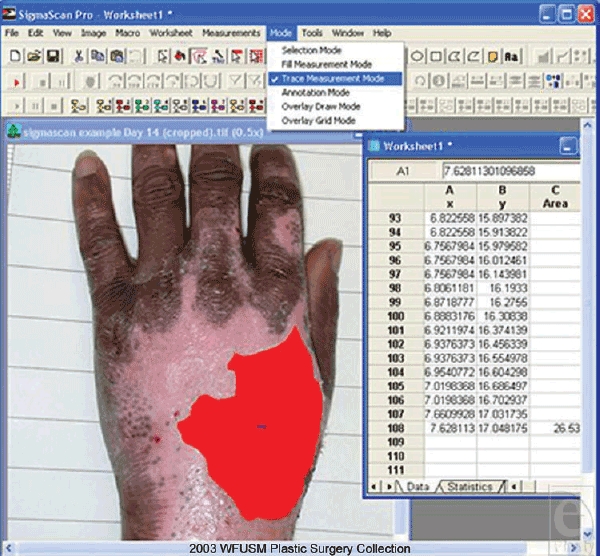
In an example of a photo obtained in the clinical study, SigmaScan planimetry is used to determine the area of a wound by outlining the area of the wound with a mouse.

The burned hands were analyzed with 2-dimensional planimetry, using SigmaScan Pro 5.0. To accomplish this, the program had to be calibrated for distance before the areas of burn could be measured. After opening the program and image, image/calibrate/distance and area were selected to open a separate window. “Two-point rescaling” was chosen and 2 points 10 cm apart, as determined by the gradations from the image backdrop, were selected. In the “old distance” box, a new value automatically appears, and in the “new distance” box, a value of “10” was entered to finish calibrating the distance. To calculate the area of the burn, measurement/measurement settings were selected to open a window. The “area” box was checked. By selecting mode/trace measurement mode, the mouse was used to outline the burn. After the borders were marked, right clicking the mouse created a Microsoft Excel type spreadsheet with the area calculated in square centimeters (Fig 4). With burns extending beyond the dorsum of the hand, the fingers were included, and the wrist crease or other consistent landmarks were used as the proximal boundary. Circumferential burns about the hand were marked to the edges of the hand on the dorsal view.

## RESULTS

Planimetry estimates from the wound models were all greater than the predetermined area. However, analysis determined SigmaScan was accurate to within 4.7% (3.4%–5.9%, 95% CI) of the actual area being measured (P < .02).

The techniques described were successfully used to evaluate an initial 20 patients in an international study involving 7 centers.^1^ Each center was able to learn consistent use of the cameras with instructions from the Web site. Adobe Photoshop normalized any color and exposure differences between images and was able to salvage the occasional inferior image (Fig 3). Individuals denying experience in photography have been able to obtain reliable and useful images for clinical evaluation and quantification of wound area.

## DISCUSSION

The use of the Internet and digital imaging is not a novel technique. It has been used in many areas of medicine,^1^–^9^ but there has been no reported method to allow for quantitative data collection from multiple institutions. In the current study, we were attempting to use the visual inspection of images obtained by digital photography to obtain quantitative estimates of burn wound size in a prospective multicenter trial of a new technique of acute burn care necessitating the development and validation of the technique.^1^

Numerous studies have investigated wounds, and they often differ in how wound area is measured. A common technique is to use some template, acetate paper, or clear plastic to trace the area of the wound. The template area can be analyzed with grid planimetry or scanned for computer-assisted digital planimetry.^10^–^13^ Langemo et al^14^ used plaster of paris to make templates of wounds and 3-dimensional stereophotogrammetry to analyze the templates.^13^ A less precise method of planimetry is diameter product where the 2 maximal perpendicular dimensions of the wound are calculated.^10^ Ioannovich et al^15^ used a computer that was able to take an image and differentiate the color of the wound from normal skin to calculate the wound area. Our decision to use SigmaScan digital planimetry was due to the need for a program that could reliably measure surface area from a Web-based photograph. Using SigmaScan is also less cumbersome and time consuming than tracing templates and making molds.

However, SigmaScan does have some errors, most of which are operator dependent. Possible problems arise when calibrating the distance or tracing the outline of area in question. These issues can be minimized with careful attention to detail and user experience. Burn edges can also be difficult to interpret, as healed epidermis is often hypopigmented and can be confused for the wound. Magnification and image enhancement with Abode Photoshop helped properly identify and mark the actual wound edges. In this study, the same experienced burn surgeons performed the wound evaluations with SigmaScan in a blinded fashion to avoid bias and interobserver variations in measurement.

Since the hand is a 3-dimensional object, using 2-dimensional planimetry presents obvious problems. In the hand model used to test the quality of our analysis, the calculated areas were usually larger than the actual area. This was because the dorsum of the hand is closer to the camera than the grids used to standardize the measurements on SigmaScan. However, the accuracy remained 4.7% within the actual area because the hand's edges are curved, so this area is underestimated with 2-dimensional planimetry, compensating for the overall overestimation. In the present study, we were evaluating changes in the same hand over time. Since it is likely that this error is a constant with each photo evaluated, it would introduce no additional error to the evaluation.

Digital photography was chosen over 35-mm photography for a number of reasons. In this Web-based multicenter study, the images were to ultimately be archived onto a computer. Digital photographs can be reproduced quickly and are easy to store and transfer, making them more convenient and time efficient.^15^,^16^ With a digital camera, an appropriate port connection or card reader is all that is required to transfer the images to the computer. Thirty-five-millimeter cameras require additional steps to archive images to the PC. The actual 35-mm film can be either made into slides or developed into photo prints. In either case, the slide/print needs to be scanned in order for digital archiving and analysis. As a general rule, one would prefer to scan slide film rather than print since the print is yet another generation removed from the original.

A full-frame 35-mm slide scanned at 2400 dpi will be about 3600 × 2400 pixels (8.6 megapixels) creating an excellent image. A 6″ × 4″ 35-mm photo print scanned at 200 dpi will be about 400 × 600 pixels (0.240 megapixels) producing a poor image.^17^,^18^ The minimum resolution acceptable to recognize details of a patient image is 0.39 megapixels,^16^ so scanning photo prints results in suboptimal PC images.

When comparing image quality between 35-mm slides scanned at high resolution and that of a digital camera, the 35-mm slide has better color and resolution, but this comes at the expense of using more storage memory. The Nikon digital image is captured as an 8-bit TIFF with 2048 × 1536 pixels (3.1 megapixels) and is stored as 9 megabytes^19^ while the 35-mm slide can be stored with 8.6 megapixels of resolution requiring 22 megabytes of storage. The improved resolution of the 35-mm film (8.6 vs 3.1 megapixels) may seem important, but Jones et al^10^ demonstrated minimal difference between in-person evaluation of hand burns versus digital images (with resolutions of 3.1 megapixels). Therefore, the 8.6-megapixel resolution from the 35-mm slide is in excess of what is needed to evaluate hand burns properly, and the excess memory requirement to store these images (22 vs 9 megabytes) only makes storage and transfer of the data more cumbersome.

With digital photography, there is one setup cost for the digital camera and memory card. This allows for unlimited photographs if the images are uploaded to a PC periodically. A camera similar to that used in this study and memory cards may readily be purchased for less than $300 at many Web sites and retailers, at the time of submission of this article for publication, examples of well-known retailers include www.buy.com, www.amazon.com, www.bestbuy.com, and www.circuitcity.com.

Adobe Photoshop 6.0 and SigmaScan are very powerful programs that add significantly to the overall cost of the study. The prices of both of these programs have slightly changed with an expected increase in functionality and features. The newest Adobe Photoshop^20^ CS3 retails for $649 and SigmaScan^21^ Pro 5.0 retails for $999.95. As discussed earlier, SigmaScan allowed accurate planimetry of images transported over the Web. Adobe Photoshop also allows for Web-transferred data to be analyzed, and it is a commonly available program with optimal editing capabilities.^16^ Of particular note, the newest version of Adobe Photoshop CS3 extended version, retailing for $999,^20^ has image measurement and analysis features that may make SigmaScan an unnecessary adjunct. In our experience, it was extremely useful in making poor images interpretable (Fig 3). The only difficulty in its use was adjusting the gray scale of each image. Because of the bending of the backdrop and angle of lights, the gray scale was not a uniform tone. Therefore, different areas on the gray backdrop had to be sampled before a satisfactory image was achieved.

In conclusion, our method of collecting images on a background with a digital camera and analysis of the burn area and appearance with SigmaScan and Adobe Photoshop, respectively, allow quantitative and qualitative analysis of the data. Currently, our institution and others are using these techniques in a study of burn wounds. Future studies monitoring the effectiveness of therapeutic treatments in Web-based multicenter trials with visual endpoints could potentially apply these methods.
